# 16th European Network of Sport Education Forum

**DOI:** 10.3390/sports12050136

**Published:** 2024-05-18

**Authors:** Louis Moustakas, Antonio Tessitore

**Affiliations:** 1 Sport, Culture and Event Management, University of Applied Sciences Kufstein, 6330 Kufstein, Austria; 2 Department of Human and Sport Science, University of Rome “Foro Italico”, 00135 Rome, Italy; antonio.tessitore@uniroma4.it

**Keywords:** sport education, social inclusion, health, coaching, policy, education, sustainable development

## Abstract

The 16th European Network of Sport Education (ENSE) Forum was held in Rome, Italy at the University of Rome ‘Foro Italico’ on 21 and 22 September 2023. The Forum was organised under the theme Sport Education for Sustainable Development: The Euro-Med Perspective and featured presentations and input from over 40 researchers, officials and policymakers. In this report, we highlight the key themes addressed at the Forum and highlight some of the notable contributions at the event.

## 1. Introduction

The European Network of Sport Education (ENSE) hosted its 16th biannual forum in Rome, Italy on 21 and 22 September 2023. ENSE, which has been active since 1989, works to create learning opportunities, skills, competencies, and qualifications in all settings for people or organisations leading, developing and supporting sport activities. Recognising the growing and pressing challenges of our era, this year’s event took place under the theme of *Sport Education for Sustainable Development: The Euro-Med Perspective*. As such, this year’s Forum offered a space for reflection and discussion on the current societal and educational challenges facing Europe and the broader Mediterranean Basin. In particular, the Forum focused on the role of sport education in a transitioning world and tackled issues such as social cohesion and intercultural dialogue, sustainability, coach education, and health. Along with panel discussions and keynote presentations, the Forum included numerous thematic scientific presentations. Overall, over 70 participants from across Europe joined the Forum and engaged with 32 presentations as well as three panel discussions. As hosts, Italy was well represented among the attendees, as were Albania, Belgium, Denmark, Germany, Greece, Hungary, Kosovo, Malta, the Netherlands, Portugal and Romania.

The first day featured an opening panel along with 23 presentations and the ENSE General Assembly. The second day included two panels, eight presentations and one film viewing. In general, the presentations featured a mix of practical projects, recently published data and ongoing projects. In the following, we will highlight a selection of themes and presentations from the Forum and conclude with some final reflections on the event. This is not a chronological depiction of the event but is rather meant to extract and reflect on some of the key ideas of the Forum. The full programme can be found at www.sporteducation.eu accessed on 20 March 2024, and the book of abstracts will be published there as well.

## 2. Sport Education and Social Inclusion

A key focus of this year’s Forum was around the role of sport education in supporting the inclusion of individuals from diverse social, cultural, educational and ability backgrounds. On day one, colleagues presented work from Italian and European projects focusing on the support and integration of individuals with disabilities within sporting contexts. Angela Magnanini, from the University of Rome ‘Foro Italico’, presented a conceptual overview of sport and social inclusion, which was then followed up by numerous project and empirical presentations. For instance, Nicole Maussier, also from ‘Foro Italico’, presented results from the Katautism project, which aims to include autistic children in the school environment through the practice of Judo and Karate, using an adapted sport education methodology [1], whereas her colleague Paolo Lucattini introduced work from the Cascina Oremo project, which focuses on offering innovative, open-sport and recreation environments for people with and without disabilities [2]. Later in day one, the focus shifted to the skills and structure needed by sport educators to support inclusion within their diverse professional contexts. Amongst others, Konstantinos Chatznikolaou of the Aristotle University of Thessaloniki presented results from the INTERCOMP project concerning the intercultural competencies of educators [3], and Louis Moustakas of the German Sport University provided conceptual input from the Sport and Social Cohesion Lab on how participatory programme approaches, such as Living Labs, could be integrated into the sport context [4]. Specific attention was also given to the key area of sport coaching, with Lisa Kalina, project manager at ENSE, presenting the first results of a scoping review to support the development of anti-bullying education for coaches [5] and Simone Ciaccioni from ‘Foro Italico’ outlining the development of a coach education course focusing on older individuals [6].

On day two, the focus on social inclusion continued, with particular attention paid to how sport education and sport programmes can support gender equality and safeguarding. Karen Petry from the German Sport University presented the results of a sport-based development intervention in Northern Iraq, highlighting how the programme helped foster improved attitudes towards gender equality [7]. Tiphaine Clerincx, from the Free University Brussels, presented the theory and development process behind an educational programme to reduce gender-based violence in sports. Further complementing this, attendees were offered a special screening of the movie *Refugee Girls*, which traces the journeys of three young refugees navigating integration, sport and education within their new lives in Barcelona and Rome (for more information, see [8]).

## 3. Sport Education and Health Promotion

Another important topic of the Forum centred around the development of tools and learning opportunities for sport educators to better support healthy lifestyles in Europe. In particular, numerous innovative online and interactive learning offers were presented. Edmond Hajrizi of the University of Business and Technology in Kosovo provided a conceptual overview of how new technologies might play a role in supporting healthy lifestyles. Susana Franco, from IP Santarem, showcased key results from the New Health 2022 project [9], which aimed to provide easy-to-obtain-and-understand knowledge and tools to support holistically healthy lifestyles. Project highlights include extensive online resources and a mobile app, as well as the development of a European-wide qualification for healthy lifestyle promoters [10]. Konstantinos Chatznikolaou from Thessaloniki took the floor again to present the concept and footage from the virtual reality game developed as part of project ViRAL. This unique project seeks to tap into the immersive and interactive opportunities offered by virtual reality (VR) to provide anti-doping education for young athletes [11]. Elsewhere, Francesca Lenzi from ‘Foro Italico’ presented a unique mobile app meant to raise awareness and participation in physical and social activity in urban environments, while Francesco Cassese and Anna Maria Mariani of the Unicusano University offered insights from the SportinForma training programme to support the healthy development of youth.

## 4. Sport Education: Skills and Policies

A last major area of the Forum concerned the pedagogical and policy structures that frame sport education and the work of sport educators in Europe. On day two, a multi-faceted panel discussion on the future of education in sport was co-chaired by Karen Petry of the German Sport University and Johan de Jong from the Hanze University of Applied Sciences. Building on themes in a related book [12], the chairs and panel discussed current trends affecting sport education in the coaching, health and social areas, and identified future directions to help support and develop the sector. This discussion was further complemented by numerous presentations throughout the Forum, with specific emphasis on issues of competence development, policy and mobility, including as concerned the fields of sport coaching, sport management and physical education.

For instance, Flavia Guidotti from San Raffaele University presented an evidence-based framework supporting a comprehensive understanding of the intertwined relationships among the knowledge, competencies, and skills needed for sport managers [13], while Gaetano Raiola of ‘Foro Italico’ discussed the issues and challenges around the mobility of coaches in Italy and across the EU.

## 5. Conclusions

The 16th ENSE Forum offered a space for a broad spectrum of discussion on the role of sport and sport education—and the sport educator—in addressing today’s many challenges, from healthy living and social inclusion to gender equality. This is a welcome development. To tap into the potential of sport, it is vital to develop well-rounded sport educators who not only have a grasp of sport or movement skills but who have a well-rounded understanding of the social, political, pedagogical and cultural factors that surround the field. The many presentations and discussions held in Rome offered crucial insights into these numerous realities and set the table for another fruitful Forum in 2025.

## Data Availability

Not applicable.
